# Subcutaneous Dirofilariosis of the Eyelid Brought to Poland from the Endemic Territory of Ukraine

**DOI:** 10.3390/pathogens12020196

**Published:** 2023-01-28

**Authors:** Beata Rymgayłło-Jankowska, Marta Ziaja-Sołtys, Beata Flis, Anna Bogucka-Kocka, Tomasz Żarnowski

**Affiliations:** 1 Department of Diagnostic and Microsurgery of Glaucoma, Medical University of Lublin, Chmielna 1 Street, 20-079 Lublin, Poland; 2 Department of Biology with Genetics, Medical University of Lublin, Witolda Chodźki 4A Street, 20-093 Lublin, Poland; 3 Clinic of General and Children’s Ophthalmology, Medical University of Lublin, Chmielna 1 Street, 20-079 Lublin, Poland

**Keywords:** *Dirofilaria repens*, humans, subcutaneous nodule, eyelid, diagnosis, Poland

## Abstract

We present the case of a 60-year-old man who was diagnosed with a subcutaneous nodule on the upper eyelid of his left eye. The patient reported multiple mosquito bites during numerous work trips to Ukraine. Histopathological examination of the nodule isolated during surgery suggested *Dirofilaria repens* infestation. The infection was brought to Poland from the territory of Ukraine. Ophthalmologists must be aware of uncommon presentations of parasitic infestations when they consider infections of the ocular adnexa.

## 1. Introduction

The mosquito-borne filaroid nematodes, *Dirofilaria immitis* (Leidy, 1856) and *Dirofilaria repens* (Raillet and Henry, 1911) primarily cause cardiopulmonary (*D. immitis*) and subcutaneous (*D. repens*) dirofilariasis in dogs [1].

*Dirofilaria repens*, i.e., a subcutaneous parasite of either dogs or cats, and foxes, occurs endemically in warm climate geographical areas on various continents. The invasion is transmitted to humans by many species of mosquitoes that are both zoophilic and anthropophilic (*Anopheles, Culex, Aedes* sp.). *Dirofilaria* nematodes are delivered before the sucking of blood commences as the mosquito probes the skin with its fascicle, at this time the L3 larva crawls out from the labellum onto the skin. It then enters the host subcutaneous tissues via the bite wound where they may migrate and or undergo maturation. Adult females of *D. repens* are usually 100 to 170 mm long and 460 to 650 µm wide, while males are smaller and usually 50 to 70 mm long and 370 to 450 µm wide [2,3]

*Dirofilaria repens* are dioecious nematodes. Humans are random hosts of *Dirofilaria* sp. and, in most of the reported cases, the infectious larvae died before the worms matured and did not develop to the fertile adult stage [4,5]. The cause of this phenomenon is the complex of *D. repens* antigens and its endosymbiont *Wolbachia* sp. generating an immune response of the host that prevents the complete development of the nematode and leads to its killing [6]. One study has observed microfilaremia in a human host [7]. It has been described that the migration of *D. repens* in tissues can last up to several weeks, months and even years after infection [8].

The process may be accompanied by such clinical symptoms as local swellings, burning or itching. *D. repens* infestations often have subconjunctival localization [9,10,11,12].

Migration of larvae through blood vessels to distal sites (e.g., male reproductive organs and central nervous system) may result in embolization. *D. repens* nematodes often affect facial tissues, especially around the eye, as well as intraocular and periocular tissues. Other *Dirofilaria* species capable of invading eye tissues are *D. immitis* and *D. tenuis.* In most cases, the diagnosis is made after the dead, damaged or live nematode is isolated from the patient’s tissue, most commonly, due to the infectious larvae undergoing some stage of development in an abnormal host before being recognized and destroyed by the immune system [13,14,15].

The probable diagnosis of human dirofilariasis caused by *D. repens* consists of identifying the presence of grooving of its cuticle with longitudinal ridges. Other species of filaria of this genus infesting humans do not have grooves. The exceptions are *Dirofilaria* sp. “hongkongensis” and *Dirofilaria ursi*, but so far no cases of their occurrence in Poland have been described [16].

## 2. Detailed Case Description

### 2.1. Clinical History

In March 2020, a 60-year-old (y.o.) man presented to the Department of Ophthalmology with a painless firm nodule localized on the left upper eyelid. Five–six months before presentation, the patient noticed a swelling of the left upper eyelid and mild ptosis. Preceding the onset of the eyelid edema, the patient had experienced a mild flu-like illness. Two–three months later, the swelling decreased significantly and the patient noticed a nodule of the upper left eyelid localized slightly temporally. There was no history of trauma, injury, or impairment of visual acuity. The patient was a professional truck driver, regularly working in the transport of goods to and from Ukraine. The patient recalled multiple mosquito bites during his business trips to Ukraine, especially at nights while sleeping in his truck in forest parking lots. 

The results of the ocular examination of the right and left eye (anterior and posterior segments) were normal, and the distant visual acuity was 1.0 in both eyes. The periocular examination of the left eye revealed a cherry-sized nodule situated slightly temporally on the left upper eyelid. The nodule was firm in consistency, moveable, non-tender, and attached to the skin but not to the underlying structures. There was no local erythema. A few days later, the nodule was completely surgically removed. 

### 2.2. Ocular Pathology

#### 2.2.1. Macroscopic Examination

**Figure 1:**
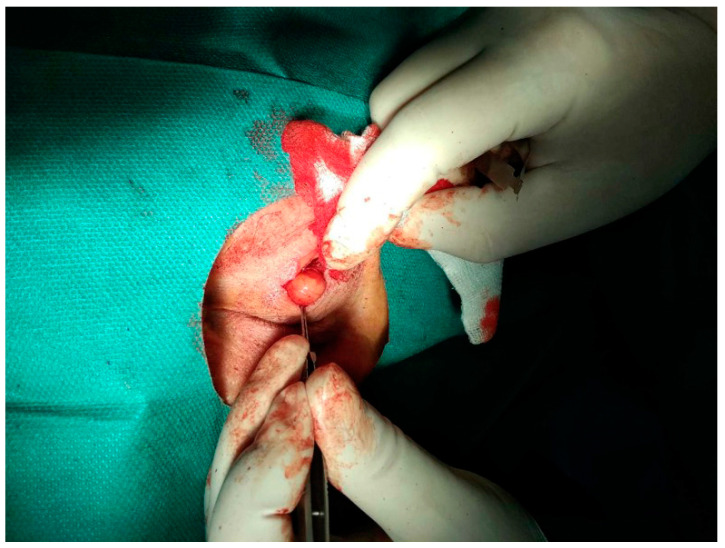
Surgical removal of the subcutaneous nodule located slightly temporally on the upper left eyelid, March 2020.

The specimen consisted of a red-brown encapsulated spherical mass measuring 1.5 × 1.5 × 1.3 cm. On dissection, the nodule was filled with serous fluid (Figure 1). The features of the nodule did not indicate a neoplastic lesion. 

#### 2.2.2. Microscopic Examination

**Figure 2:**
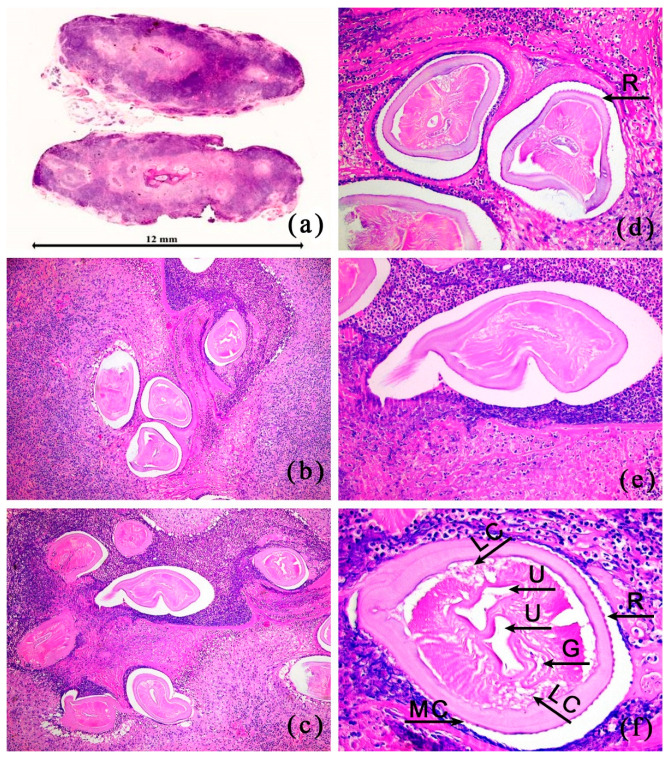
(**a**) Cross-section of the subcutaneous nodule surgically isolated from the patient. Hematoxylin and eosin staining. The bar in the photo indicates the magnification; (**b**,**c**) Cross sections of female *D. repens* localized in the subcutaneous nodule isolated from the patient, (40× magnification); (**d**) the typical external cuticular ridges are well visible (R), (**e**) *D. repens* cross-section visible at a higher magnification (200×); (**f**) A closer view of the *D. repens* individual section; cuticular ridges (R), uterus (U), gut (G), multilayered cuticle (MC), lateral chords (LC), (400× magnification).

At a low magnification, multiple granulomas were observed with cross-sections of a worm/parasite present inside. At a higher magnification, the worm had a thick multilayered cuticle with longitudinal ridges, lateral chords, a paired uterus, and an alimentary tract. Around the worm, an inflammatory reaction was observed consisting of macrophages, epithelioid cells, lymphocytes, plasma cells, neutrophils, and numerous eosinophils. All these features suggested a dirofilariasis infestation by *D. repens* (Figure 2).

**Figure 3:**
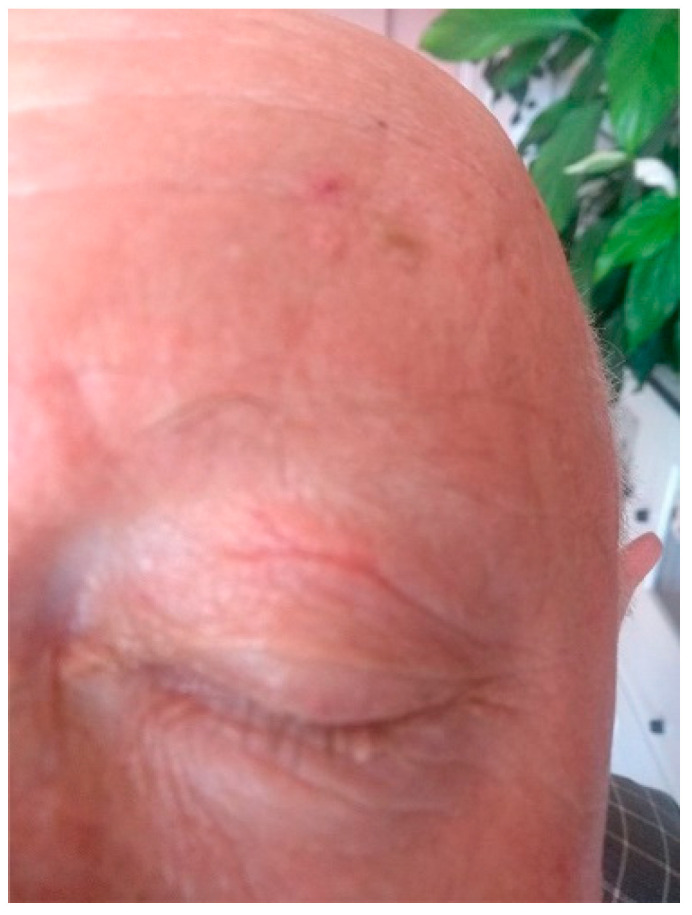
Healed post-operative scar on the upper eyelid of the left eye, May 2020.

The post-operative course was uneventful. The last patient follow-up visit took place in May 2020. The patient presented with a totally healed post-operative scar on the upper left eyelid (Figure 3). 

## 3. Discussion

Currently, it is believed that the distribution area of the filarial nematode, a parasite of animals and humans, *D. repens*, is one of the largest in Europe [6]. The main reason for the increase in the incidence of *Dirofilaria* infections among humans is climate change leading to warming and the extension of Mediterranean weather conditions to northern Europe. The consequence of this change is an increase in the number of vectors of parasites, mosquitoes, capable of surviving and developing in the new temperature conditions. The development of world tourism, international business trips and a large number of domesticated dogs and cats are also factors contributing to the increase in the number of cases of dirofilariasis among humans [17,18]. Experimental infections of mosquitoes carried out in Ukraine, relevant to Poland, revealed the development of *D. repens* to the L3 stage within 13–14 days at 18–28 °C after ingestion an infectious blood meal [19]. In recent years, dirofilariasis has spread to Central and Eastern European countries, such as Ukraine, Poland, and Slovakia as well as Northern Europe and the Baltic States: Lithuania and Estonia, as shown by the latest data [20,21].

The first cases of dirofilariasis with *D. repens* as the etiological agent in humans were diagnosed in Poland in 2007 [22]. Since all the infected patients had visited areas with endemic dirofilariasis, there was no evidence for their autochthonous origin. The first three cases of autochthonous infections in Poland were detected in 2010 [9]. In 2019, for the first time in the world, a case of a patient from Poland who was diagnosed with elbow bursitis caused by *D. repens* was described [23]. 

Humans are unusual hosts for parasites. Nevertheless, infestations in humans can cause subcutaneous nodules, local swelling through subcutaneous migration of the worm, and even severe clinical manifestations affecting various organs (e.g., the brain or lungs) [24,25]. The most common are ocular and periocular infestations, which are found particularly during the migratory phase of the parasite. The nematode may be located subconjuctivally (as mentioned above) as well as in periocular tissues (eyelids, orbit) [26,27,28]. Eye symptoms, including local pain, bulging of the eye, double vision, edema of the eyelids and the conjunctiva, redness, foreign body sensation, and visual impairment, were observed in the course of ocular manifestations [29].

*D. repens* does not usually cause severe symptoms. Our 60-year-old patient was asymptomatic—the upper eyelid nodule was painless. He was successfully treated by the total removal of the nodule. Pharmacotherapy is not recommended in humans. Surgery is the treatment of choice. The treatment is often a result of suspicion of malignancy or diagnosis of ocular dirofilariasis, where the removal of the entire parasite during surgery is the only effective treatment [16,17].

Currently, no indirect immunological methods are used in the diagnosis of dirofilariasis in humans, because the microfilarial stage that induces immune reactions is rare; hence, antibodies directed against filaria are not detected in most patients. In addition, the absence of microfilariae in the blood prevents the use of molecular methods to identify the etiological agent of infections based on DNA samples of the parasite. Therefore, histopathological testing of nodules isolated from patients is commonly used [4]. When subcutaneous dirofilariasis is considered, costly cancer diagnosis (computer tomography (CT), magnetic resonance imaging (MRI), etc.) can be avoided. However, in areas endemic to dirofilariasis, imaging techniques have proven useful in differentiating nematode granulomas from tumor-like lesions. As reported by some authors, detection of antibodies against *Wolbachia* surface proteins is perhaps one of the possibilities to improve the serodiagnosis of dirofilariasis, since this bacteria is not found in other helminths besides filaria [30].

In the medical interview, the patient recalled multiple mosquito bites while he was driving his truck to Ukraine. In Ukraine, Poland’s eastern neighbor, dirofilariasis has been known for a long time. The first cases of *D. repens* infestation were recorded over a hundred years ago among dogs (Petropavlovskij 1904), while the first human infestation was reported in 1927 (Skrabin et al., 1930) [8]. 

Nowadays, in European Union countries, there is no obligation to register *Dirofilaria* infestations. Epidemiological data for these countries come from scientific publications. Of the European countries, a mandatory system of registration of dirofilariasis cases in humans exists only in Ukraine (since 1975). Thanks to these data, it is known that the number of cases in Ukraine is still growing, and the cases are recorded from all over the country [8].

By analyzing and summarizing these data, the *Dirofilaria* infection in our patient was found to be a result of travelling to an endemic region of *D. repens.* Due to the mass influx of migrants from Ukraine to Poland since February 2022, the frequency of *D. repens* infections diagnosed in our country may increase significantly.

## 4. Conclusions

*Dirofilariasis* should be considered in the differential diagnosis of non-inflammatory and inflammatory mass lesions of periocular tissues. Ophthalmologists must be aware of the uncommon presentations of parasitic infestations when they consider infections of the ocular adnexa.

## Data Availability

Not applicable.
